# SLC-0111, an inhibitor of carbonic anhydrase IX, attenuates hepatoblastoma cell viability and migration

**DOI:** 10.3389/fonc.2023.1118268

**Published:** 2023-01-26

**Authors:** Katja Eloranta, Marjut Pihlajoki, Emmi Liljeström, Ruth Nousiainen, Tea Soini, Jouko Lohi, Stefano Cairo, David B. Wilson, Seppo Parkkila, Markku Heikinheimo

**Affiliations:** ^1^ Pediatric Research Center, Children’s Hospital, Helsinki University Hospital, University of Helsinki, Helsinki, Finland; ^2^ Department of Pathology, University of Helsinki and Helsinki University Hospital, Helsinki, Finland; ^3^ Xentech, Evry, Evry, France; ^4^ Istituto di Ricerca Pediatrica, Padova, Italy; ^5^ Champions Oncology, Hackensack, NJ, United States; ^6^ Department of Pediatrics, Washington University School of Medicine, St. Louis Children’s Hospital, St. Louis, MO, United States; ^7^ Department of Developmental Biology, Washington University School of Medicine, St. Louis, MO, United States; ^8^ Faculty of Medicine and Health Technology, Tampere University, Tampere, Finland; ^9^ FICAN Mid, Tampere University, Tampere, Finland; ^10^ Fimlab Ltd, Tampere University Hospital, Tampere, Finland; ^11^ Faculty of Medicine and Health Technology, Center for Child, Adolescent, and Maternal Health Research, Tampere University, Tampere, Finland

**Keywords:** carbonic anhydrase IX, hepatoblastoma, pediatric oncology, tumor hypoxia, targeted therapy

## Abstract

**Background:**

In response to hypoxia, tumor cells undergo transcriptional reprogramming including upregulation of carbonic anhydrase (CA) IX, a metalloenzyme that maintains acid-base balance. CAIX overexpression has been shown to correlate with poor prognosis in various cancers, but the role of this CA isoform in hepatoblastoma (HB) has not been examined.

**Methods:**

We surveyed the expression of CAIX in HB specimens and assessed the impact of SLC-0111, a CAIX inhibitor, on cultured HB cells in normoxic and hypoxic conditions.

**Results:**

CAIX immunoreactivity was detected in 15 out of 21 archival pathology HB specimens. The CAIX-positive cells clustered in the middle of viable tumor tissue or next to necrotic areas. Tissue expression of *CAIX* mRNA was associated with metastasis and poor clinical outcome of HB. Hypoxia induced a striking upregulation of CAIX mRNA and protein in three HB cell models: the immortalized human HB cell line HUH6 and patient xenograft-derived lines HB-295 and HB-303. Administration of SLC-0111 abrogated the hypoxia-induced upregulation of CAIX and decreased HB cell viability, both in monolayer and spheroid cultures. In addition, SLC-0111 reduced HB cell motility in a wound healing assay. Transcriptomic changes triggered by SLC-0111 administration differed under normoxic vs. hypoxic conditions, although SLC-0111 elicited upregulation of several tumor suppressor genes under both conditions.

**Conclusion:**

Hypoxia induces CAIX expression in HB cells, and the CAIX inhibitor SLC-0111 has *in vitro* activity against these malignant cells.

## 1 Introduction

Hepatoblastoma (HB) is a rare pediatric liver malignancy with an incidence of 2.16 per million person-years (1). Approximately 80% of cases are diagnosed before the age of three years (2). Although the etiology of HB is not well understood, its morphology and molecular landscape suggest an embryonal origin (3–5). Most HB cases are sporadic, but certain congenital disorders such as Beckwith-Wiedemann syndrome and familial adenomatous polyposis are risk factors for HB development (3). HB treatment entails surgical resection or liver transplantation combined with pre- and post-operative administration of cisplatin, carboplatin, and doxorubicin (6). This approach has improved the 5-year overall survival (OS) rate to greater than 80% (7), though patients with metastases or relapsed/refractory disease have significantly lower survival rates, emphasizing the need for novel treatment strategies (8).

Solid tumors often contain hypoxic regions due to an imbalance between microvascularization and rapid growth (9). The hypoxic microenvironment is associated with cancer progression and treatment failure (10). Cancer cells adapt to low oxygen tension *via* hypoxia inducible factor 1α (HIF1α) mediated responses including upregulation of carbonic anhydrase (CA) IX (11). CAs are evolutionary conserved metalloenzymes catalyzing reversible hydration of CO_2_ to ${\text{HCO}}_{3}^{-}$ and H^+^ (12). In humans, fifteen CA isoforms have been identified, three of which lack catalytic activity (13). Transmembrane CAIX is a tumor associated CA isoform with restricted expression in healthy tissue (14). In fetal liver, scattered CAIX-positive hepatocytes have been reported, but postnatal CAIX immunoreactivity in liver is limited to bile duct cells (15–17).

High CAIX expression has been linked to enhanced cell survival, high proliferation rate, increased motility/invasion, and chemoresistance in a wide range of tumors including breast, lung, and oral cancers (18–20). CAIX plays a pivotal role in maintaining acid-base balance in tumors (21). While intracellular acidification reduces tumor cell survival and can be utilized to kill cancer cells, an acidic extracellular milieu supports tumor progression (22). CAIX drives both neutralization of the intracellular compartment and acidification of the tumor microenvironment, promoting an aggressive cancer phenotype (23). Consequently, CAIX is an attractive therapeutic target. A small molecule inhibitor of CAIX, SLC-0111, has completed a phase 1 clinical trial for treatment of advanced solid tumors; no significant dose-limiting toxicities were encountered (24).

Herein, we characterize CAIX expression in archival HB specimens and use cell culture models to study the impact of SLC-0111 on this malignancy. We show that hypoxia induces CAIX expression in HB cells and that SLC-0111 has considerable *in vitro* activity against this cancer.

## 2 Materials and methods

### 2.1 Patient samples

Samples were acquired from the Helsinki Biobank at Helsinki University Hospital. Informed written consent was collected at the time of sample deposit. The study was approved by an ethics committee at Helsinki University Hospital (HUS/3319/2018) and was performed in accordance with Finnish bylaws. Tumor samples (n=21) were obtained from HB patients treated at Children’s Hospital, Helsinki University Hospital between January 1, 1990 and December 31, 2017. Sampling was performed during surgical resection or liver transplantation (after pre-operative chemotherapy). Normal liver (NL) control samples were collected from organ donors (n=3).

### 2.2 Immunohistochemistry

Five µm sections of formalin-fixed paraffin embedded tumor specimens were deparaffinized and immunostained with a monoclonal anti-human CAIX antibody (M75) (25). Immunoperoxidase staining was performed using an automated Lab Vision Autostainer 480 (LabVision Corporation, Fremont, CA, USA) and Power Vision+ Poly-HRP Immunohistochemistry kit reagents (ImmunoVision Technologies Co). The staining protocol included the following steps: (a) rinsing in wash buffer; (b) treatment in 3% H_2_O_2_ in ddH_2_O for five minutes and rinsing with wash buffer; (c) blocking with cow colostrum diluted 1:2 in Tris-buffered saline (TBS) containing 0.05% Tween-20 for 30 minutes and rinsing in wash buffer; (d) incubation with 1:100 diluted M75 for 30 minutes; (e) rinsing in wash buffer three times for five minutes each; (f) incubation with poly-HRP-conjugated anti-mouse IgG for 30 minutes and rinsing in wash buffer three times for five minutes each; (g) incubation in DAB (3,3`-diaminobenzidine tetrahydrochloride) solution (one drop of DAB solution A and one drop of DAB solution B in 1 ml of ddH_2_O) for six minutes and rinsing in ddH_2_O; (h) CuSO_4_ treatment for five minutes to enhance the signal and rinsing in ddH_2_O; (i) treatment with hematoxylin for one minute; (j) rinsing with ddH_2_O. All steps were performed at room temperature (RT). Imaging was performed using 3DHISTECH Panoramic 250 FLASH II digital slide scanner at Genome Biology Unit (Research Programs Unit, Faculty of Medicine, University of Helsinki Biocenter, Helsinki, Finland).

### 2.3 Clinical data

Raw microarray data of gene expression and clinical data from 53 HB tissue samples and 14 noncancerous liver tissue samples were acquired from the Gene Expression Omnibus (GEO) database of the National Center for Biotechnology Information (NCBI) (http://www.ncbi.nlm.nih.gov/geo/), accession number GSE131329. Microarray data were analyzed with Chipster software (https://chipster.rahtiapp.fi/) (26) using the normalization tool for Affymetrix gene arrays (27, 28). Statistical tests were conducted using the “Two group tests” tool (empirical Bayes as test and Benjamini-Hochberg as p-value adjustment method) (29). Differences in the distribution of continuous variables were assessed using the Mann-Whitney U test. Statistical significance was set to p-value < 0.05. Analyses were conducted with IBM SPSS Statistics version 28.0 (IBM Corp., Armonk, NY, USA).

### 2.4 Cell lines and maintenance

The human HB cell line HUH6 was purchased from the Japanese Collection of Research Bioresources Cell Bank (Osaka, Japan). HB cell lines established from patient-derived xenografts (PDX; HB-303 and HB-295) were obtained from XenTech (Evry, France). HUH6 cells were maintained in Dulbecco’s modified Eagle medium (DMEM)-GlutaMAX (glucose: 1 g/l) supplemented with 10% fetal bovine serum (FBS), 100 U/ml penicillin, and 100 µg/ml streptomycin (all from Gibco, Stockholm, Sweden). HB-303 and HB-295 cells were cultured in Advanced DMEM/F12 medium (Gibco) supplemented with 8% FBS, 100 U/ml penicillin, and 100 µg/ml streptomycin, and 20 µM of Y-27632 (SelleckChem, Houston, TX, USA). Cells were routinely maintained at +37°C in a humidified incubator with 5% CO_2_. In this study, these conditions represent normoxia (21% O_2_) (30). Hypoxic conditions were generated utilizing the XVivo incubation system (partial pressures: 94% N_2_, 1% O_2_, 5% CO_2_) (BioSpherix, Parish, NY, USA). All cell lines were authenticated through short tandem repeat profiling.

### 2.5 SLC-0111 and cisplatin treatments

Carbonic anhydrase IX/XII inhibitor SLC-0111 (alias: U-104) was purchased from SelleckChem (catalog no. S2866) and dissolved in sterile DMSO as a 10 mM stock solution. Further dilutions were prepared in adequate cell culture medium. Normal cell culture medium supplemented with DMSO was utilized as a control treatment. Incubations were performed for 48 h if not otherwise stated, and the medium was not changed during the incubations.

### 2.6 RNA and protein extraction

RNA and protein extraction was performed with Nucleospin RNA/Protein Mini extraction kit (Macherey-Nagel, Düren, Germany) following the manufacturer’s instructions.

### 2.7 RNA sequencing and data processing

HUH6 cells were cultured under normoxic or hypoxic conditions and treated with 100 µM of SLC-0111 or vehicle. RNA was extracted after 48 h incubation. Prior to sequencing, RNA concentration, quality, and integrity were assessed by the Biomedicum Functional Genomics Unit (Helsinki Institute of Life Science and Biocenter Finland, University of Helsinki, Finland) using the TapeStation system (Agilent, Glostrup, Denmark). RNA libraries were prepared applying polyA selection, and Illumina compatible cDNA libraries were constructed by GENEWIZ (Leipzig, Germany). Subsequently, samples were sequenced on Illumina NovaSeq 6000 yielding 2x150bp paired end reads (GENEWIZ). An RNA sequencing dataset containing 11 HB patient samples and 11 NL samples was obtained from the GEO database of NCBI (accession number: GSE151347) (31, 32). FastQC tool was utilized to control quality of the reads (33). Sequenced reads were mapped to human reference genome hg38 using HISAT2 aligner (34). Reads per gene were counted with HTseq (35). To analyze differentially expressed genes (DEGs), edgeR2 tool was employed (36). Cut-off values were set to fold change lg_2_ +/-0.8 and Benjamini-Hochberg adjusted p-value <0.05. Preprocessing of data and DEG analysis was carried out with Chipster (26). Gene Ontology (GO) analysis was performed with Enrichr (37, 38). R packages tidyverse and ggplot2 were used for data visualization in R (v. 4.0.3).

### 2.8 Real-time quantitative PCR (RT-qPCR)

RNA was reverse transcribed using the iScript cDNA Synthesis Kit (Bio-Rad, Hercules, CA, USA) following the manufacturer’s instructions. RT-qPCR was carried out with a CFX384 thermocycler instrument (Bio-Rad), and PowerUP SYBR Green Master Mix (Thermo Fisher Scientific) was used for gene amplification. Relative gene expression was assessed using the 2^-ΔΔCT^ method (39). Geometric mean of *ACTB* and *PPIG* expression served as a reference. Primer sequences were: *ACTB;* GCGTGACATCAAAGAGAAGC (forward), AGGATTCCATACCCAAGAAGG (reverse); *CAIX* GCCTTTGCCAGAGTTGACGA (forward), TCTGAGCCTTCCTCAGCGAT (reverse); *PPIG* CAATGGCCAACAGAGGGAAG (forward), CCAAAAACAACATGATGCCCA (reverse).

### 2.9 Western blotting

Proteins (10 µg) were separated by electrophoresis using Mini-Protean TGX Stain-Free Gels (Bio-Rad, Hercules, CA, USA) Next, proteins were transferred onto polyvinyl fluoride membrane. Blocking was performed with 5% non-fat milk in TBS. Primary antibody incubations were performed at +4°C for overnight (anti-human CAIX rabbit IgG at a of dilution 1:1500; NB100-417, Novus Biologicals, Littleton, CO, USA). Secondary antibody incubation was carried out at RT for 1 h (1:10,000; #111-035-144, Jackson ImmunoResearch, West Grove, PA, USA). Protein bands were illuminated using the Enhanced Chemiluminescence detection kit (Amersham ECL reagent; GE Healthcare, Barrington, IL, USA). Quantification was performed with Image Lab Software 6.0 (Bio-Rad). CAIX band intensities were normalized to the amount of total protein in the corresponding lane using stain-free technology (37).

### 2.10 Wound healing assay

Ibidi-treated cell culture inserts (3-well in µ-dish; Ibidi, Munich, Germany) were used to generate wounds. Cells were seeded into inserts in high density 24 h prior to the experiment. Before treatment initiation with vehicle or 100 µM of SLC-0111, inserts were removed, and cells were washed with phosphate buffered saline (PBS) to eliminate debris. Wounds were imaged at treatment outset (0 h) and after 20 h with an Eclipse TS100 microscope supplemented with the DS-Fi1 digital imaging system (Nikon, Tokyo, Japan). Wounds (16 images/insert) were analyzed with ImageJ software to calculate the percentage of wound closure. The following formula was used: Wound closure (%) = ((W_0_ – W_t_)/W_0_) x 100 (W_0_ = Wound area at 0 h and W_t_ = Wound area at 20 h).

### 2.11 Spheroid cultures

Cells were seeded at a density of 2000 cells/well into 96-well ultra-low attachment plates (PerkinElmer, Waltham, MA, USA) and cultured without disturbance for 48 h at +37°C in a humified incubator with 5% CO_2_. After the establishment period, cells were incubated with vehicle or increasing concentrations of SLC-0111 for 48 h.

### 2.12 Viability measurements

Cell viability (ATP concentration) was assessed with the ATPLite™ 2D or 3D monitoring system (PerkinElmer) following manufacturer’s instructions. Luminescence was measured with a GloMax microplate reader (Promega, Madison, WI, USA).

### 2.13 Immunofluorescence

HUH6 and HB-303 cells (100 000 cells/well) were grown in 4-well chamber slides coated with collagen I for 24 h. Cells were fixed with 4% paraformaldehyde. Non-specific binding was blocked with UltraVision Protein Block solution (Thermo Scientific, Fremont, CA, USA). Next, cells were incubated with primary antibody at room temperature for 1 h (NB100-417 human anti-rabbit CAIX at 1:1000 dilution, Novus Biologicals, Littleton, CO, USA). Secondary antibody incubation was performed with goat anti-rabbit IgG (H+L) AlexaFluor 647 (1 h, room temperature) at 1:800 dilution (A32733, Invitrogen, Carlsbad, CA, USA). Images were captured with a Zeiss Axio Imager M2 (objective: EC Plan Neofluar 40 X/0.75 Ph2 M27) (Carl-Zeiss, Oberkochen, Germany).

Spheroids were fixed with chilled 100% methanol for 20 minutes at RT. Following washes with PBS, 0.1% Triton-X was utilized to permeabilize the cells. Nonspecific binding was blocked with UltraVision Protein Block Solution (Thermo Fisher). Primary antibody incubation (anti-human CAIX rabbit IgG, at a dilution of 1:100; NB100-417, Novus Biologicals) was performed at RT for 1.5 h. Subsequently, spheroids were incubated with secondary antibody (anti-rabbit IgG (H+L) AlexaFluor 647, at dilution 1:200; A32733, Thermo Fisher) at RT for 1 h. Hoechst (at dilution 1:2000; #62249, Thermo Fisher) was used for nuclear staining. Opera Phenix High Content Screening System was employed to capture images (Perkin Elmer). Imaging was performed in the High Content Imaging and Analysis unit (FIMM, University of Helsinki).

### 2.14 Target prediction analyses

Potential bioactive targets of SLC-0111 were assessed with SwissTargetPrediction (http://www.swisstargetprediction.ch/) (40) and SUPERpred (https://prediction.charite.de) (41) online tools.

### 2.15 Statistical analysis

Cell experiments were conducted in triplicate. Statistical analyses were carried out with GraphPad Prism (v. 8.4.2; San Diego, CA, USA). Student’s t-test or one-way ANOVA followed with Tukey’s test were utilized to assess statistical significance depending on the experimental setting. p-value < 0.05 was considered significant.

## 3 Results

### 3.1 CAIX protein expression in clinical HB samples

**Figure 1:**
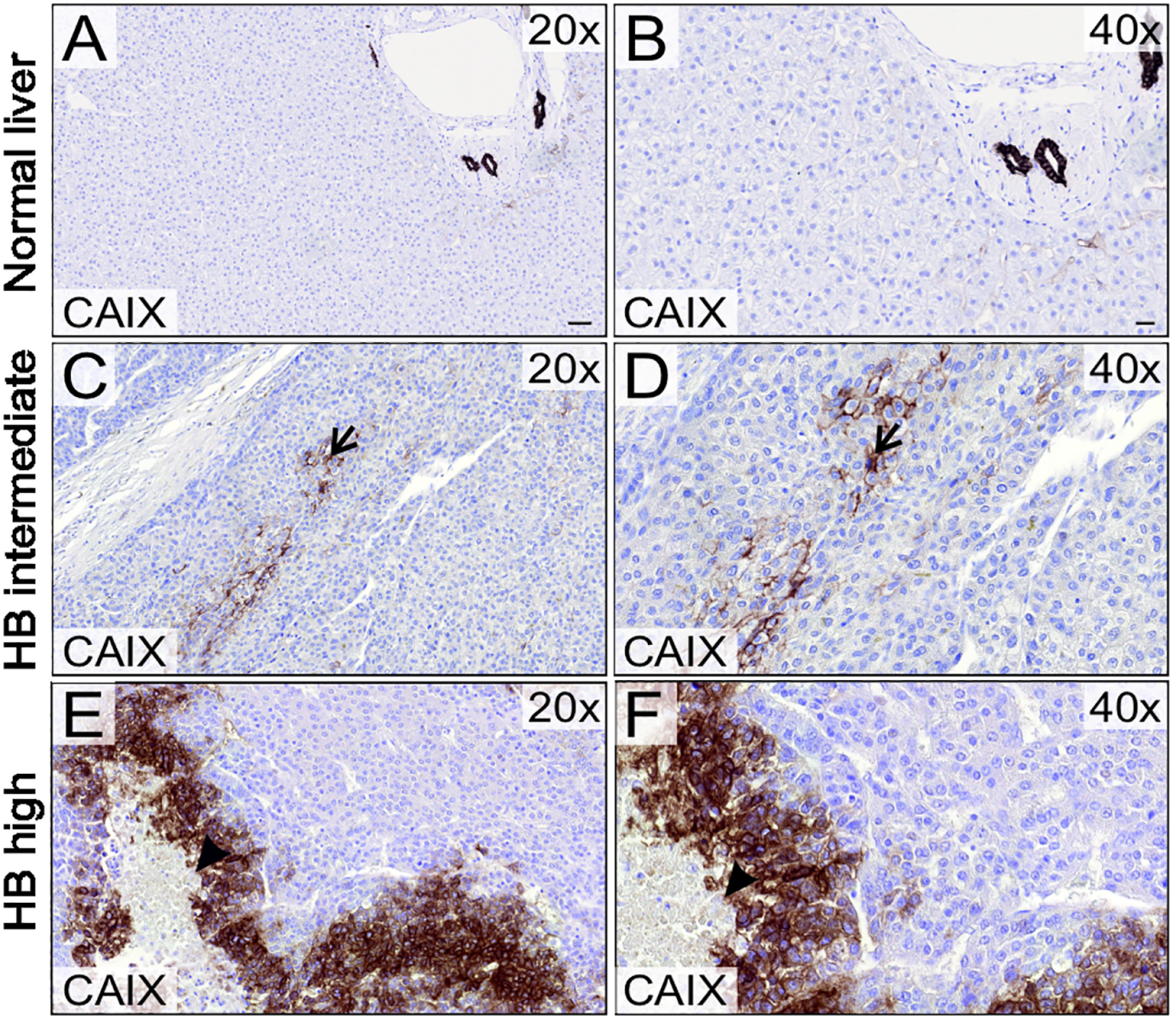
CAIX expression in HB patient samples and normal liver. CAIX expression was restricted to bile duct cells in normal liver tissue **(A, B)**. 9/21 HB tumor samples demonstrated intermediate **(C, D)** and 6/21 high CAIX immunoreactivity **(E, F)**. CAIX expression localized to small clusters in the middle of viable HB tissue (arrow, **C, D**) or adjacent to necrotic areas (arrowhead, **E, F**). Scale bars: 50 µm **(A)** and 20 µm **(B)**.

We analyzed 21 specimens of HB (11 male, 10 female) in the Helsinki Biobank. The median patient age at surgery was 3.18 years (0.23-10.83 years). Patient characteristics, treatments, and CAIX expression status are summarized in **Table 1**. Three pediatric donor liver samples (age 2.0-8.2 years) were used as normal controls. Consistent with previous studies, in healthy liver CAIX immunostaining was restricted to bile duct cells (**Figures 1A, B**) (16, 17). Over 70% of the HB specimens demonstrated CAIX immunoreactivity; 9/21 had intermediate (**Figures 1C, D**) expression, and 6/21 had high CAIX expression (**Figures 1E, F**). CAIX staining was predominantly membranous in both the HB and healthy liver samples (**Figures 1A–F**). Within HB specimens, CAIX-positive cells were grouped in small clusters in the middle of viable tissue (**Figures 1C, D**) or adjacent to necrotic areas (**Figures 1E, F**), regions presumed to be hypoxic due to limited blood supply.

**Table 1:** HB patient characteristics and CAIX expression status.

Patient	Age at sampling (years, age group)	Sex (Male/Female)	PRETEXT	Histology	Surgery	Chemo	CAIX (-/+/++)
HB1	>7	M	3, P	Fetal, epithelial	TX	SIOPEL-4	+
HB2	3-7	M	4, B	Fetal	TX	SIOPEL-4, sorafenib, vincristine, etoposide	+
HB3	3-7	F	3, M	n/a	TX	SIOPEL-4, sorafenib, vincristine, fluorouracil	++
HB4	3-7	M	3, A1	Fetal, epithelial	TX	SIOPEL-4	+
HB5	3-7	F	3, V, E	Fetal, embryonal	TX	SIOPEL-4	–
HB6	1-3	M	2, A1	Fetal, epithelial	Resection	SIOPEL-4	+
HB7	3-7	M	Unknown	Epithelial, macrotrabecular	TX	n/a	–
HB8	>7	M	M	Fetal, well-differentiated	TX	n/a	+
HB9	<1	F	3, A1	Fetal, epithelial	Resection	SIOPEL-4	–
HB10	>7	F	4, E1, H1	Fetal, epithelial	TX	SIOPEL-4	++
HB11	<1	M	2, A1	Fetal, epithelial	TX	Cisplatin	+
HB12	1-3	M	2	Mixed epithelial/ mesenchymal	TX	SIOPEL-4	–
HB13	3-7	F	4, M	Fetal, epithelial	TX	SIOPEL-4	+
HB14	1-3	M	4, M, V	Embryonal, mixed	TX	SIOPEL-4	++
HB15	3-7	M	2, H1	Fetal, epithelial	Resection	SIOPEL-4	++
HB16	3-7	F	2, P2	Embryonal	TX	SIOPEL-4	–
HB17	1-3	M	3	Fetal, epithelial	TX	SIOPEL-4	–
HB18	3-7	F	3, M	Fetal, epithelial	Resection	n/a	++
HB19	1-3	F	4	Fetal, epithelial, well differentiated	TX	n/a	+
HB20	1-3	F	3	Epithelial, embryonal and fetal	Resection	n/a	+
HB21	1-3	F	2	Mixed epithelial/ mesenchymal, teratoid features	Resection	n/a	++

TX=liver transplantation.

++ = high CAIX expression.

+ = intermediate CAIX expression.

- = no CAIX expression.

n/a = data not available.

### 3.2 CAIX mRNA expression in HB correlates with poor clinical outcome

**Figure 2:**
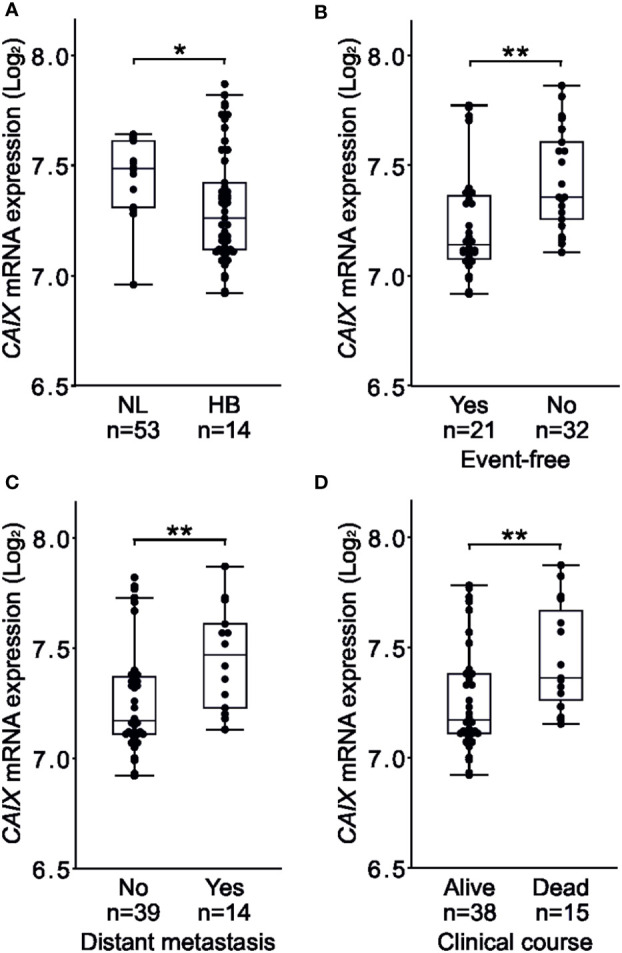
High *CAIX* expression associates with events, distant metastases, poor overall survival in HB. Total *CAIX* expression was higher in normal liver compared to HB samples **(A)**. Occurrence of events associated with higher *CAIX* expression **(B)**. Patients with distant metastasis demonstrated higher *CAIX* expression compared to those with no metastasis **(C)**. Poor overall survival was linked to elevated *CAIX* expression **(D)**. Dots represent individual samples, the box represents the interquartile range, the whiskers represent the 1st and 4th quartile and the line inside the box is the median. *p-value < 0.05, **p-value < 0.01.

In the Helsinki cohort, all 5 cases of metastatic HB demonstrated CAIX immunoreactivity (**Table 1**), suggesting that CAIX expression correlates with advanced disease. We used a larger patient cohort [GSE131329, a dataset containing 53 HB and 14 normal liver samples] to compare *CAIX* mRNA expression with three clinical variables – occurrence of an unfavorable event, metastasis, and overall survival. Total *CAIX* expression was higher in normal liver (median 7.485, [IQR 7.308–7.613]) than in HB samples (median 7.260, [IQR 7.115–7.470]) (**Figure 2A**), likely a reflection of CAIX expression in the biliary epithelium of normal tissue. The occurrence of any event was associated with higher *CAIX* expression (median 7.360 [IQR 7.245-7.640]) than an event-free disease course (median 7.145 [IQR 7.075-7.376]) (**Figure 2B**). Patients with distant metastases had higher *CAIX* expression (median 7.470 [IQR 7.223-7.638]) than those without metastases (median 7.170 [IQR 7.110-7.380]) (**Figure 2C**). Poor overall survival was associated with elevated *CAIX* expression (HB median 7.230 [IQR 7.162-7.360] vs. normal liver median 7.105 [IQR 6.999-7.170]) (**Figure 2D**).

### 3.3 Hypoxia induces CAIX expression in cell models of HB

**Figure 3:**
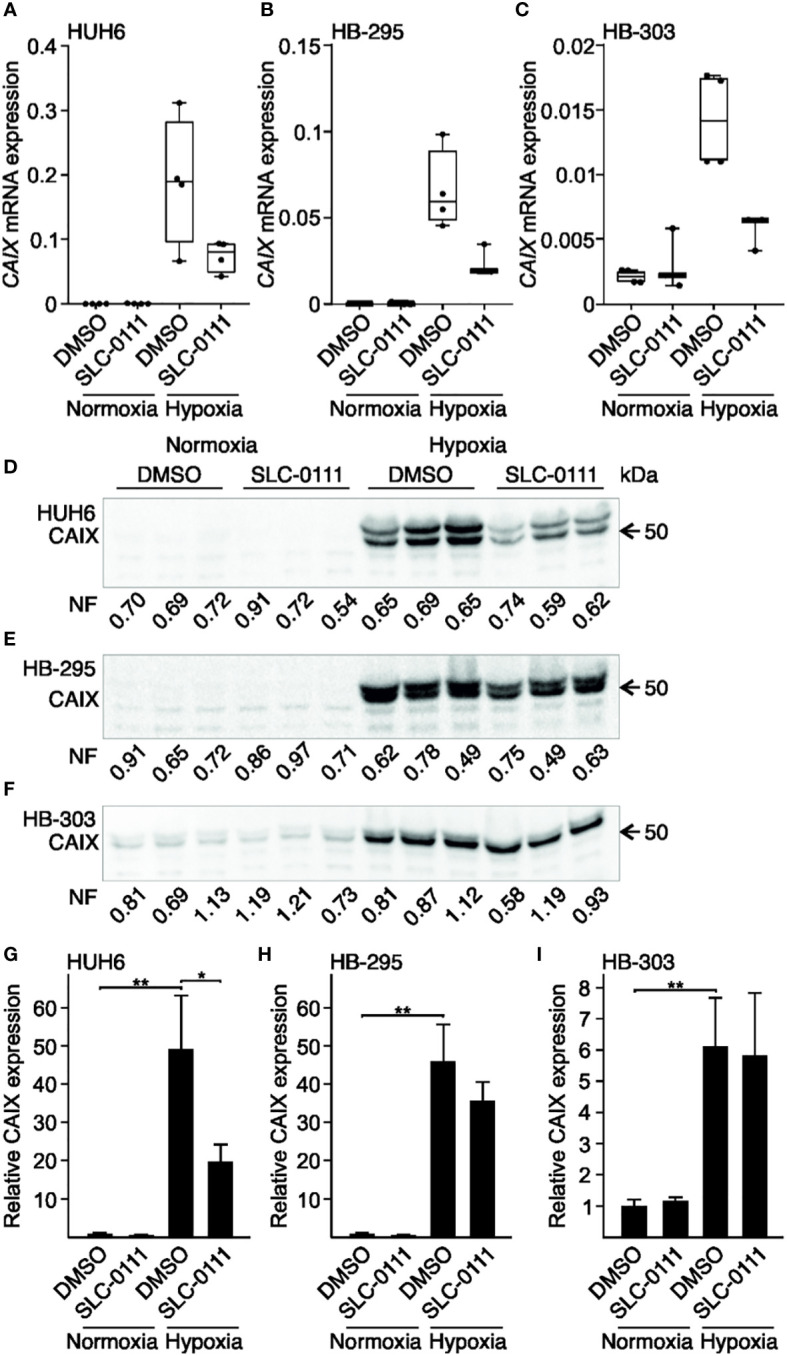
Hypoxia-induced expression of CAIX in HB cell lines is attenuated by SLC-0111. *CAIX* mRNA expression was negligible under normoxic condition in HUH6 **(A)**, HB-295 **(B)**, and HB-303 **(C)** cells. In response to hypoxia, *CAIX* mRNA expression drastically increased in all cell lines **(A–C)**. SLC-0111 treatment decreased *CAIX* mRNA expression 40-60% under hypoxia **(A–C)**. CAIX protein levels were significantly higher in cells grown under hypoxic compared to normoxic conditions in all cell models **(E–I)**, and in hypoxic HUH6 cells CAIX expression significantly decreased following SLC-0111 treatment **(D, G)**. Bar plots reflect the mean of three independent experiments ± RSD. Band intensity was normalized to total protein expression in each lane. Normalization factor (NF) describing the amount of total protein in lane relative other lanes is given beneath the bands. *p-value < 0.05, **p-value < 0.01. SLC-0111 = 100 µM.

We used cell culture models to investigate whether low oxygen tension induces CAIX expression in HB. The immortalized human HB cell line HUH6 or the PDX-derived cell lines HB-295 and HB-303 were cultured in a hypoxic chamber or normoxic incubator for 48 h. All three HB cell lines demonstrated little or no baseline *CAIX* mRNA expression when cultured under normoxia (**Figures 3A**–C). *CAIX* mRNA expression was markedly upregulated in hypoxic cells compared to normoxic controls, with increases of 740-fold in HUH6 (**Figure 3A**), 165-fold in HB-295 (**Figure 3B**), and 6.7-fold in HB-303 cells (**Figure 3C**). Similarly, hypoxia induced 6- to 50-fold increases in CAIX protein levels (**Figures 3D–I**).

Next, we assessed the impact of 100 µM SLC-0111 on CAIX mRNA and protein expression under normoxic and hypoxic conditions. In normoxia, *CAIX* mRNA expression remained invariant after SLC-0111 treatment in all three cell models (**Figures 3A–C**). HB cells cultured under hypoxia and treated with SLC-0111 demonstrated a 40-60% reduction in *CAIX* mRNA expression compared to vehicle treated control cells (**Figures 3A–C**). Following SLC-0111 treatment, levels of CAIX protein decreased significantly in hypoxic HUH6 cells but not in HB-295 or HB-303 cells (**Figures 3D–I**).

### 3.4 SLC-0111 treatment attenuates HB cell viability in monolayer and spheroid cultures

**Figure 4:**
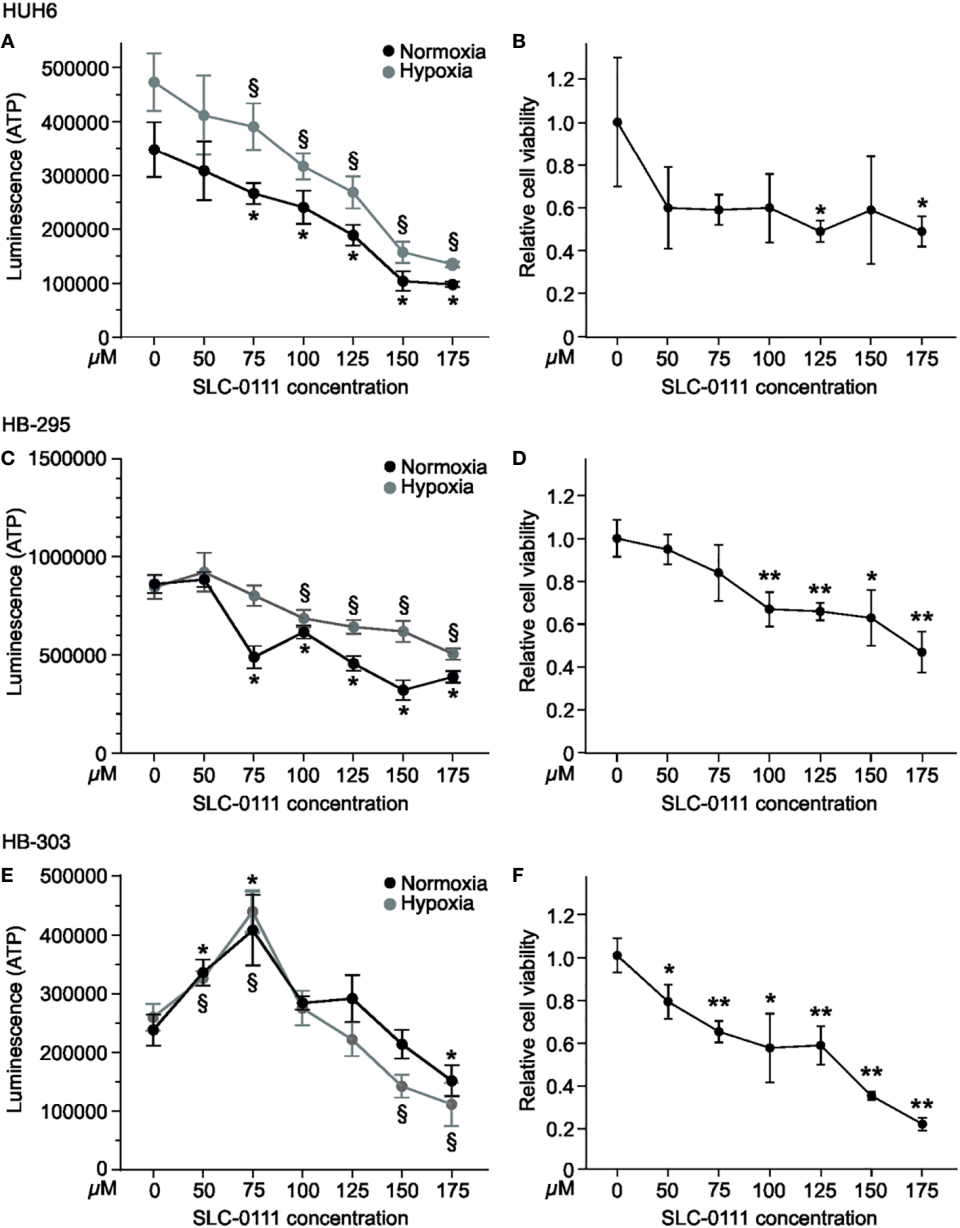
SLC-0111 decreases cell viability of HB monolayers and spheroids. To assess cell viability, ATP levels were measured after SLC-0111 treatment. HUH6 cell viability decreased dose-dependently both in normoxia and hypoxia **(A)**. HUH6 spheroids showed significantly decreased cell viability at SLC-0111 concentrations of 125 and 175 µM **(B)**. In HB-295 cells the reduction in cell viability was significant at concentrations between 75-175 µM under normoxia and between 100-175 µM under hypoxia **(C)**. In HB-295 spheroids the viability was decreased at concentrations of 100-175 µM **(D)**. The response of HB-303 to SLC-0111 differed from other models. Cell viability increased at concentrations of 50-100 µM and decreased at concentrations of 125-175 µM **(E)**. In HB-303 spheroids the viability was decreased with all concentrations **(F)**. *and ^§^p-value < 0.05, **p-value < 0.01 (compared to corresponding control).

To explore the effects of CAIX inhibition on HB cell survival in monolayer and spheroid cultures, we measured ATP concentrations, a surrogate for cell viability, following exposure of cells to increasing amounts of SLC-0111. In monolayer cultures, HUH6 cell viability decreased in a dose-dependent manner both in normoxia and hypoxia (**Figure 4A**). As with HUH6 cells, the impact of SLC-0111 on the viability of HB-295 monolayer cultures was more pronounced in normoxic than hypoxic conditions (**Figure 4C**). HB-303 had a dissimilar response to SLC-0111 than the other two models; cell viability increased with doses of 50-100 µM and with doses of 125-175 µM a modest decrease in viability was observed (**Figure 4E**).

The spatiotemporal distribution of oxygen in solid cancers cannot fully be mimicked in monolayer cell cultures. Instead, spheroids more closely resemble the 3-dimensional architecture of solid tumors, as oxygen levels differ for cells exposed directly to growth medium vs. those located in the inner parts of spheroids (42). To further assess the effects of SLC-0111 on HB cells, we used spheroids cultured in normoxia. SLC-0111 elicited a decrease in viability in all three HB spheroid models (**Figures 4B, D, F**). We also noticed spontaneous expression of CAIX in HB spheroids under normoxic conditions, whereas the cells grown in 2D showed negligible CAIX expression (**Supplementary Figure 1**).

### 3.5 HB cell motility is impaired by SLC-0111 treatment

**Figure 5:**
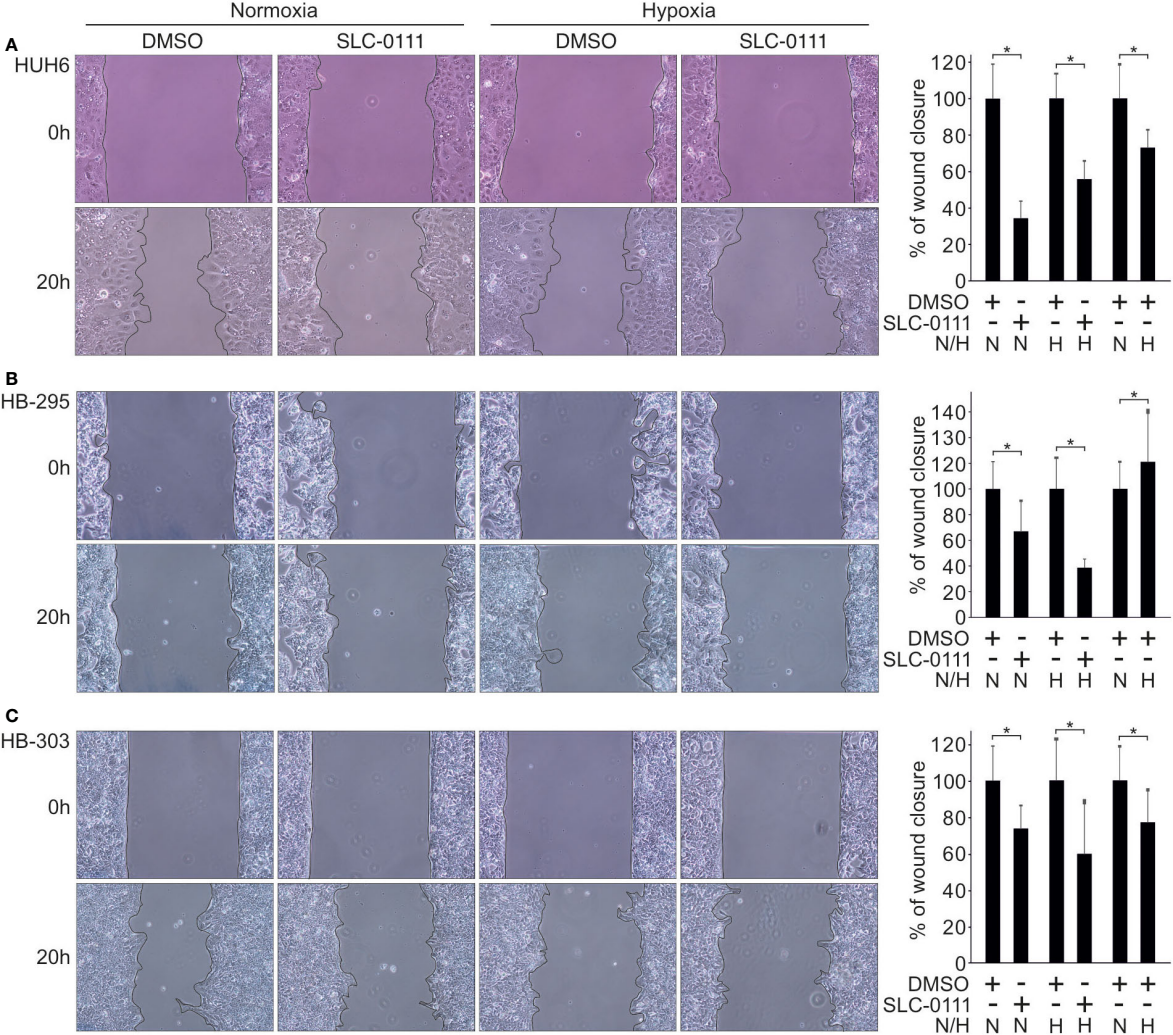
SLC-0111 treatment decreases cell motility in HB cell models. After 20 h of SLC-0111 treatment, migration rate was significantly reduced in HUH6 **(A)**, HB-295 **(B)**, and HB-303 **(C)** cells in both normoxic and hypoxic conditions. Histograms show the percentage of wound closure relative to DMSO treated control. Bar plots are presented as relative values of mean of three independent experiments ± RSD. *p-value < 0.05. SLC-0111 = 100 µM. N, normoxia; H, hypoxia.

Several studies have reported decreased cell motility after pharmacological inhibition of CAIX or silencing of the *CAIX* gene (43–45). In our HB cell models, migration rates decreased significantly after 20 h treatment with 100 µM SLC-0111 compared to control cells both under normoxic and hypoxic conditions (**Figures 5A–C**). SLC-0111 had the most drastic effect on migration in HUH6 cells, wherein motility decreased approximately 70% in normoxia and 40% in hypoxia after 20 h of SLC-0111 treatment (**Figure 5A**). Hypoxia increased the migratory capacity of HB-295 cells compared to normoxic control cells, and SLC-0111 reduced motility in both normoxia and hypoxia (**Figure 5B**). A modest reduction in migration was observed in HB-303 cells treated with SLC-0111. In these cells the decrease was approximately 30% in normoxia and 35% in hypoxia compared to corresponding vehicle treated controls (**Figure 5C**).

### 3.6 Transcriptomic changes induced by SLC-0111 diverge in normoxic and hypoxic conditions

**Figure 6:**
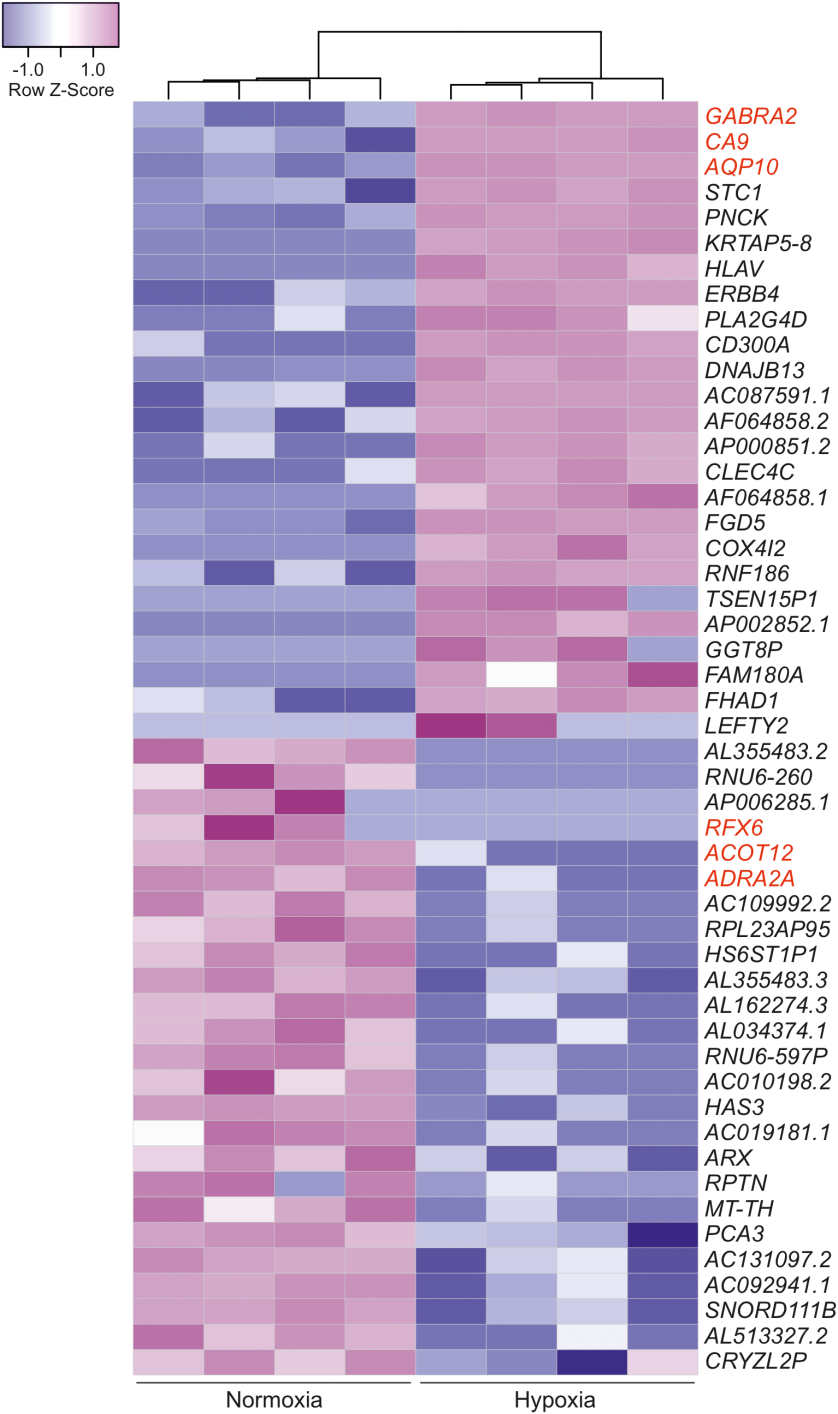
Hypoxia-induced transcriptomic alterations in HUH6 cells. Heatmap of the 25 most downregulated and the 25 most upregulated differentially expressed genes in RNA sequencing analysis performed for cells grown under normoxic or hypoxic conditions sorted by logFC. The three most up- and downregulated protein coding genes are highlighted in red color.

As noted above, SLC-0111 decreased viability and motility in HB cells even under normoxic conditions when CAIX expression was undetectable or extremely low, suggesting that the drug may have CAIX-independent effects. To explore transcriptomic changes induced by SLC-0111 treatment, we performed RNA sequencing analysis for HUH6 cells treated with 100 µM SLC-0111 for 48 h under either normoxia or hypoxia. First, we assessed global gene expression alterations triggered by hypoxia compared to baseline expression in normoxia. A total of 2876 DEGs were observed of which 2155 genes were upregulated and 721 were downregulated (**Supplementary Figure 2**; **Supplementary Table 1**). The three most upregulated protein coding genes were gamma-aminobutyric acid receptor subunit alpha-2 (*GABRA2*), *CAIX*, and aquaporin 10 (*AQP10*) (**Figure 6**; **Supplementary Table 2**). Regulatory factor X6 (*RFX6*), acyl-CoA thioesterase 12 (*ACOT12*), and adrenoceptor alpha 2A (*ADRA2A*) were the most downregulated protein coding genes under hypoxia in comparison to normoxia (**Figure 6**; **Supplementary Table 2**).

**Figure 7:**
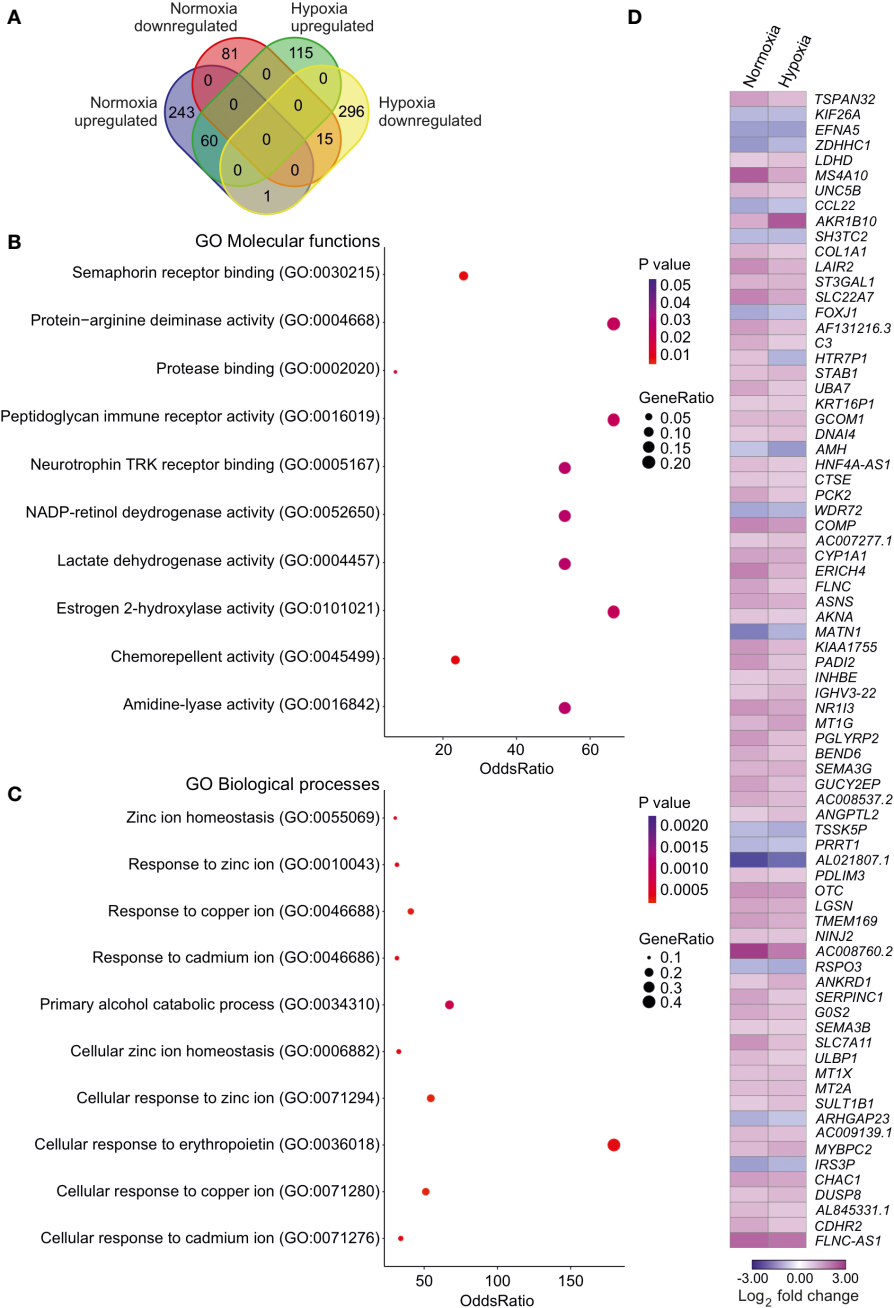
Impact of SLC-0111 on gene expression in normoxic and hypoxic HUH6 cells. RNA sequencing analysis identified 243 upregulated and 81 downregulated genes after SLC-0111 treatment in normoxia **(A)**. In hypoxia the corresponding numbers were 115 upregulated and 296 downregulated **(A)**. 60 genes were upregulated and 15 downregulated both in normoxia and hypoxia **(A)**. One gene was upregulated in normoxia but downregulated in hypoxia **(A)**. Enriched molecular functions in overlapping genes **(B)**. Enriched biological processes in overlapping genes **(C)**. Heatmap of overlapping differentially regulated genes in hypoxia and normoxia **(D)**.

Next, we assessed the effects of SLC-0111 on the HUH6 cell transcriptome. In normoxia, we observed 304 upregulated genes and 96 downregulated genes after SLC-0111 treatment (**Supplementary Table 1**). Under hypoxic conditions, SLC-0111 induced upregulation of 175 genes and downregulation of 312 genes (**Supplementary Table 1**). Altogether, 76 genes were differentially expressed in both normoxic and hypoxic HUH6 cells treated with SLC-0111 (**Figure 7D**). Of these 76 genes, 15 genes were downregulated both in normoxia and hypoxia, 60 genes were upregulated in both conditions, and one gene was differentially regulated in hypoxia and normoxia (**Figure 7A**; **Supplementary Table 3**). Molecular functions associated with these overlapping genes included semaphorin binding, protein-arginine deaminase activity, and protease binding (**Figure 7B**). Metal ion related biological processes were highly overrepresented in SLC-0111 treated cells (**Figure 7C**). We also characterized overlaps in genes dysregulated in HB patient samples and expression alterations caused by SLC-0111 in HUH6 cells. A total of 7411 DEGs (**Supplementary Table 1**) were noted in HB vs NL, and 35 of these genes were also differentially expressed in HUH6 cells following SLC-0111 administration (**Supplementary Table 1**). SLC-0111 treatment of HUH6 cells caused upregulation of 20 genes that were downregulated in HB tumor tissue (**Table 2**), including the tumor suppressor genes *MT1G, MT1X, MT2A, OTC, PCK2, PGLYRP2, SERPINC1*, and *NR1I3*. Three genes (*FOXJ1, PRRT1*, and *TSSK5P*) were downregulated in SLC-0111 treated HUH6 cells and upregulated in HB tumor tissue (**Table 2**).

**Table 2:** Overlaps in genes dysregulated in HB patient samples and expression alterations caused by SLC-0111 in HUH6 cells.

Symbol	Gene	Up/Downregulated (HB tissue vs. NL)	Up/Downregulated (SLC-0111 vs. DMSO)
ENSG00000125730	C3	↓	↑
ENSG00000128965	CHAC1	↓	↑
ENSG00000140465	CYP1A1	↓	↑
ENSG00000129654	FOXJ1	↑	↓
ENSG00000123689	G0S2	↓	↑
ENSG00000229005	HNF4A-AS1	↓	↑
ENSG00000139269	INHBE	↓	↑
ENSG00000214856	KRT16P1	↓	↑
ENSG00000166816	LDHD	↓	↑
ENSG00000146166	LGSN	↓	↑
ENSG00000125144	MT1G	↓	↑
ENSG00000187193	MT1X	↓	↑
ENSG00000125148	MT2A	↓	↑
ENSG00000276980	NA	↓	↑
ENSG00000143257	NR1I3	↓	↑
ENSG00000036473	OTC	↓	↑
ENSG00000100889	PCK2	↓	↑
ENSG00000161031	PGLYRP2	↓	↑
ENSG00000204314	PRRT1	↑	↓
ENSG00000117601	SERPINC1	↓	↑
ENSG00000008513	ST3GAL1	↓	↑
ENSG00000010327	STAB1	↓	↑
ENSG00000227473	TSSK5P	↑	↓

↓ = gene downregulated, ↑ = gene upregulated.

### 3.7 Target prediction analysis for SLC-0111

To identify other potential targets for SLC-0111, we performed *in silico* target prediction analysis with two online tools (SwissTargetPrediction and SUPERpred). We combined the common predicted targets from both tools (**Table 3**). In addition to CAIX and CAXII, SLC-0111 had high expected probability of binding CAII (**Table 3**). Other identified targets included histone deacetylase (HDAC) 3, thymidylate synthase (TYSY), nuclear factor NF kappa-B inhibitor kinase alpha (CHUK), mammalian target of rapamycin (mTOR), cyclin dependent kinases (CDKs) 1/2/4/5, and phosphatidylinositol 3-kinases PK3CA, PK3CB, and PK3CG (**Table 3**).

**Table 3:** Predicted bioactive targets of SLC-0111.

Uniprot ID	Target	Target Class	Swiss Target Prediction (probability)	SUPERpred (probability)
O43570	CAXII	Lyase	0.99283030414	1.0
Q16790	CAIX	Lyase	0.99283030414	1.0
P00918	CAII	Lyase	0.99283030414	1.0
P00915	CAI	Lyase	0.0978745343258	0.98
P54132	BLM	Enzyme	0.0978745343258	0.98
Q00535	CDK5	Kinase	0.0978745343258	0.9
O15379	HDAC3	Eraser	0.0978745343258	0.89
P17948	VGFR1	Kinase	0.0978745343258	0.87
P10721	KIT	Kinase	0.0978745343258	0.85
P24864	CCNE1	Kinase	0.0978745343258	0.83
P04818	TYSY	Transferase	0.0978745343258	0.83
P36888	FLT3	Kinase	0.0978745343258	0.726
P24941	CDK2	Kinase	0.0978745343258	0.72
P08235	MCR	Nuclear receptor	0.0978745343258	0.72
P06493	CDK1	Other cytosolic protein	0.0978745343258	0.7
P23219	PGH1	Oxidoreductase	0.0978745343258	0.67
P04629	NTRK1	Kinase	0.0978745343258	0.67
P42345	MTOR	Kinase	0.0978745343258	0.67
P42338	PK3CB	Enzyme	0.0978745343258	0.64
P48736	PK3CG	Enzyme	0.0978745343258	0.62
O15111	CHUC	Kinase	0.0978745343258	0.62
O00444	PLK4	Kinase	0.0978745343258	0.61
P53667	LIMK1	Kinase	0.0978745343258	0.6
P11802	CDK4	Kinase	0.0978745343258	0.6
P42336	PK3CA	Enzyme	0.0978745343258	0.6
P40763	STAT3	Transcription factor	0.0978745343258	0.58
P45984	MK09	Kinase	0.0978745343258	0.57
Q9HAZ1	CLK4	Kinase	0.0978745343258	0.54
P49759	CLK1	Kinase	0.0978745343258	0.51
P35218	CAH5A	Lyase	0.0978745343258	0.5

## 4 Discussion

Hypoxia triggers metabolic reprogramming in tumor cells, resulting in decreased intracellular pH levels (46). To counter this acidic stress, cancer cells induce the expression of CAIX (11). In various malignancies, CAIX expression associates with advanced disease and treatment failure, underscoring its potential as a biomarker and treatment target (37–41). We found that CAIX is expressed in most HB samples and is associated with unfavorable clinical outcome.

Our findings echo studies of adult liver cancer, wherein high CAIX expression has been linked to treatment resistance, recurrence, and unfavorable outcome (47, 48). Huang et al. reported a diffuse perinecrotic localization of CAIX in hepatocellular carcinoma (HCC) tissues (48). Similarly, we observed CAIX immunoreactivity in perinecrotic regions and in small clusters in the middle of viable tumor tissue in HB specimens. Cancer stem cells (CSCs) facilitating tumorigenicity and metastasis are thought to reside in specific niches within tumors, including perinecrotic regions (49). Of note, CAIX expression has been suggested to support CSC survival in PDX-models of cervical and breast cancer (50, 51).

Upregulation of CAIX expression has been observed in numerous cancer cell lines in response to hypoxia (52–56). In keeping with these studies, we found that CAIX expression was strongly upregulated at the mRNA and protein levels in HB cell models exposed to hypoxic conditions, while its baseline expression in normoxia was extremely low. Treatment with SLC-0111 abrogated hypoxia-induced *CAIX* mRNA expression in all three HB cell lines studied but caused a notable decrease in CAIX protein level in only one cell line. This may be explained by the fact that CAIX protein and mRNA expression were measured at the same timepoint. Owing to protein turnover rates, it may take longer to see a decrease in protein levels compared to RNA levels.

SLC-0111 is a ureido-sulfonamide inhibitor of CA that has been reported to target hypoxia-induced CAIX and CAXII with a high selectivity (57–59). A multitude of novel SLC-0111 analogues have recently been developed to inhibit these cancer-associated enzymes with even better selectivity compared to the classical compound (60). Since SLC-0111 acts mechanistically as an inhibitor of CAIX enzymatic activity (61), it was surprising to observe a drastic impact on *CAIX* mRNA levels in HB cells with low basal CAIX expression in the present study. A similar reduction in hypoxia-induced *CAIX* mRNA expression after SLC-0111 treatment was observed in breast cancer cells (45). Based on these findings it is possible that inhibition of CAIX activity has a negative regulatory effect on its transcription.

Tumor cell motility is a prerequisite for metastasis. Multiple studies have demonstrated an association between increased migratory or invasive capability and high CAIX expression in cancer (19, 62–65). Consistent with those reports, the motility of HB cells was reduced when CAIX function was inhibited with SLC-0111. Mechanistically, CAIX has been shown to interact with cell adhesion proteins, matrix metalloproteinases, integrins, and ion exchangers to facilitate migration and invasion (63, 66–68). Interactome studies are required to clarify which proteins are co-operating with CAIX in HB cells.

Interestingly, we noticed that SLC-0111 attenuated HB cell viability and motility when there was no observable CAIX expression, suggesting that there may be alternative targets for this drug. SLC-0111 is an efficient nanomolar inhibitor of CAIX and CAXII (69). At micromolar concentrations, SLC-0111 also inhibits CAI and CAII, consistent with our target prediction analysis (57). In metastatic lung, colorectal, and breast cancer models, combination therapy with SLC-0111 and the HDAC inhibitor SAHA has demonstrated higher potency than these agents as monotherapy (70). Moreover, this multi-drug treatment associated with increased p53 and histone H4 acetylation (70). Our target prediction analyses suggested that SLC-0111 may interact with HDAC3. SLC-0111 has potential to act as an epigenetic modifier and may potentiate HDAC inhibitors partially by targeting the very same proteins. We also observed enrichment of cell cycle regulation related proteins (CDK1/2/4/5) in predicted targets of SLC-0111. This may be one of the mechanisms how SLC-0111 reduces cell viability in normoxia and should be validated in the future. Further investigations are needed to understand SLC-0111 mechanisms of action in the absence of CAIX expression in HB as well as other tumor types.

SLC-0111 treatment triggered distinct patterns of gene expression in normoxic vs. hypoxic HUH6 cells. This suggests that the mechanism of action of SLC-0111 may be environment-dependent. Notably, we found that SLC-0111 enhanced expression of eight genes (*MT1G*, *MT1X*, *MT2A*, *OTC*, *PCK2*, *PGLYRP2*, *SERPINC1*, and *NR1I3*) under both normoxic and hypoxic conditions. Each of these genes has been shown to be epigenetically silenced or deactivated in HB or other liver malignancies, and restoring expression was associated with improved prognosis, reduced cell viability, and/or decreased metastatic capacity (71–77).

Conversely, SLC-0111 attenuated expression of *FOXJ1* in HUH6 cells under normoxia and hypoxia. Overexpression of *FOXJ1* has been linked with poor prognosis and increased proliferation rate in HCC (78). Based on these transcriptomic changes and earlier studies, we propose that SLC-0111 may act as an epigenetic modifier activating tumor suppressor genes and downregulating oncogenes in addition to functioning as a CAIX inhibitor. This mechanism could explain the drastic impact of SLC-0111 on HB cell viability and motility in the absence of observable CAIX expression. More investigations are needed to delineate the exact effectors.

To date, one clinical trial of SLC-0111 has been reported. In that Phase 1 study, no objective responses were observed in adults with advanced solid tumors, but 2 out of 17 heavily pre-treated patients had stable disease for up to 24 weeks (24). It must be emphasized that confirmed CAIX tissue expression was not used as an inclusion criterion for that study. There is also a Phase Ib clinical trial on the efficacy of SLC-0111 in combination with gemcitabine in CAIX-positive pancreatic cancer patients (79). These and future trials will hopefully identify patients who may benefit from SLC-0111 treatment. We suggest that the role of SLC-0111 as a potential epigenetic regulator of tumor suppressor genes should be considered when planning future clinical trials. Pediatric clinical trials are needed to confirm the safety of SLC-0111 in this population.

One limitation of our study is that the impact of SLC-0111 on HB was not examined *in vivo*. Another shortcoming is that several of the *in vitro* experiments were conducted in monolayer cultures which do not fully recapitulate oxygen gradients in tumor tissue.

**Figure 8:**
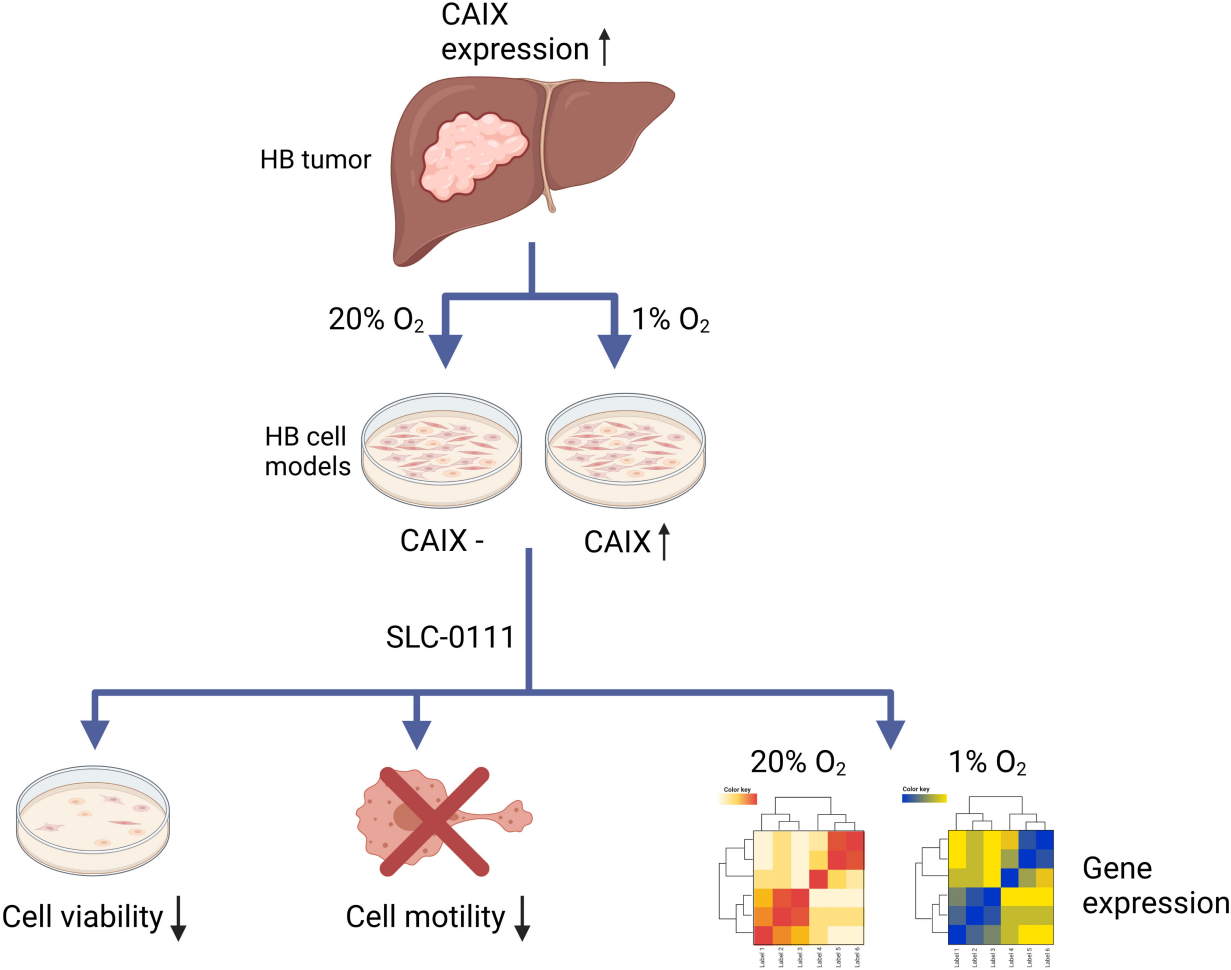
Schematic illustration of the findings.

The key findings of this study are summarized in **Figure 8**. All in all, CAIX is expressed in the majority of HBs and may have potential as a prognostic marker. In HB cell culture models, hypoxia induces *CAIX* expression, and the CAIX inhibitor SLC-0111 reduces HB cell survival and motility. Our results also suggest that SLC-0111 may have CAIX-independent effects. We speculate that SLC-0111 administration may restore expression of tumor suppressor genes in HB *via* epigenetic mechanisms.

## Data availability statement

The datasets presented in this study can be found in online repositories. The names of the repository/repositories and accession number(s) can be found below: https://www.ncbi.nlm.nih.gov/. GSE185937.

## Ethics statement

The studies involving human participants were reviewed and approved by Helsinki University Hospital institutional ethics committee. Written informed consent to participate in this study was provided by the participants’ legal guardian/next of kin.

## Author contributions

KE, MP, SP, and MH: conceptualization and research design. KE, MP, EL, RN, TS, JL, DW, SP and MH: acquisition, analysis, or interpretation of data. SC: establishing and providing PDX cell models. MP: Preparing the final Figures. KE: writing the first draft. KE, MP, EL, RN, TS, JL, SC, DW, SP, and MH: reviewing and editing. KE, MP, EL, RN, TS, JL, SC, DW, SP, and MH: final approval of the manuscript version to be published. All authors contributed to the article and approved the submitted version.

## References

[1] Feng J Polychronidis G Heger U Frongia G Mehrabi A Hoffmann K. Incidence trends and survival prediction of hepatoblastoma in children: A population-based study. Cancer Commun (2019) 39. doi: 10.1186/s40880-019-0411-7 PMC6813130 31651371

[2] Haeberle B Rangaswami A Krailo M Czauderna P Hiyama E Maibach R. The importance of age as prognostic factor for the outcome of patients with hepatoblastoma: Analysis from the children’s hepatic tumors international collaboration (CHIC) database. Pediatr Blood Cancer (2020) 67:e28350. doi: 10.1002/pbc.28350 32383794

[3] Spector LG Birch J. The epidemiology of hepatoblastoma. Pediatr Blood Cancer (2012) 59:776–9. doi: 10.1002/pbc.24215 22692949

[4] Armengol C Cairo S Fabre M Buendia MA. Wnt signaling and hepatocarcinogenesis: The hepatoblastoma model. Int J Biochem Cell Biol (2011) 43:265–70. doi: 10.1016/j.biocel.2009.07.012 19646548

[5] Nagae G Yamamoto S Fujita M Fujita T Nonaka A Umeda T. Genetic and epigenetic basis of hepatoblastoma diversity. Nat Commun (2021) 12:5423. doi: 10.1038/s41467-021-25430-9 34538872 PMC8450290

[6] Aronson DC Czauderna P Maibach R Perilongo G Morland B. The treatment of hepatoblastoma: Its evolution and the current status as per the SIOPEL trials. J Indian Assoc Pediatr Surg (2014) 19:201–7. doi: 10.4103/0971-9261.142001 PMC4204244 25336801

[7] McAteer JP Goldin AB Healey PJ Gow KW. Surgical treatment of primary liver tumors in children: Outcomes analysis of resection and transplantation in the SEER database. Pediatr Transplant (2013) 17:744–50. doi: 10.1111/petr.12144 23992390

[8] Hou JY Yeh TC Huang TH Sheu JC Liu HC. A retrospective study of clinical features and outcome in patients with refractory or recurrent hepatoblastoma: A single institution experience. Pediatr Neonatol (2021) 62:400–5. doi: 10.1016/j.pedneo.2021.03.018 33967009

[9] Vaupel P Harrison L. Tumor hypoxia: causative factors, compensatory mechanisms, and cellular response. Oncologist (2004) 9:4–9. doi: 10.1634/theoncologist.9-90005-4 15591417

[10] Hoückel M Vaupel P. Tumor hypoxia: Definitions and current clinical, biologic, and molecular aspects. JNCI J Natl Cancer Inst (2001) 93:266–76. doi: 10.1093/jnci/93.4.266 11181773

[11] Takacova M Kajanova I Kolarcikova M Lapinova J Zatovicova M Pastorekova S. Understanding metabolic alterations and heterogeneity in cancer progression through validated immunodetection of key molecular components: a case of carbonic anhydrase IX. Cancer Metastasis Rev (2021) 40:1035–53. doi: 10.1007/s10555-021-10011-5 PMC8825433 35080763

[12] Aspatwar A Tolvanen MEE Barker H Syrjanen L Valanne S Purmonen S. Carbonic anhydrases in metazoan model organisms: Molecules, mechanisms, and physiology. Physiol Rev (2022) 102:1327–83. doi: 10.1152/physrev.00018.2021 35166161

[13] Aggarwal M Boone CD Kondeti B McKenna R. Structural annotation of human carbonic anhydrases. J Enzyme Inhib Med Chem (2013) 28:267–77. doi: 10.3109/14756366.2012.737323 23137351

[14] Pastorek J Pastoreková S Callebaut I Mornon JP Zelník V Opavský R. Cloning and characterization of MN, a human tumor-associated protein with a domain homologous to carbonic anhydrase and a putative helix-loop-helix DNA binding segment. Oncogene (1994) 9:2877–88. 8084592

[15] Kallio H Pastorekova S Pastorek J Waheed A Sly WS Mannisto S. Expression of carbonic anhydrases IX and XII during mouse embryonic development. BMC Dev Biol (2006) 6:22. doi: 10.1186/1471-213X-6-22 16719910 PMC1526727

[16] Liao SY Lerman MI Stanbridge EJ. Expression of transmembrane carbonic anhydrases, CAIX and CAXII, in human development. BMC Dev Biol (2009) 9:22. doi: 10.1186/1471-213X-9-22 19291313 PMC2666674

[17] Pastorekova S Parkkila S Parkkila AK Opavsky R Zelnik V Saarnio J. Carbonic anhydrase IX, MN/CA IX: Analysis of stomach complementary DNA sequence and expression in human and rat alimentary tracts. Gastroenterology (1997) 112:398–408. doi: 10.1053/gast.1997.v112.pm9024293 9024293

[18] Chu CY Jin YT Zhang W Yu J Yang HP Wang HY. CA IX is upregulated in CoCl2-induced hypoxia and associated with cell invasive potential and a poor prognosis of breast cancer. Int J Oncol (2016) 48:271–80. doi: 10.3892/ijo.2015.3253 26648580

[19] Yang JS Lin CW Hsieh YH Chien MH Chuang CY Yang SF. Overexpression of carbonic anhydrase IX induces cell motility by activating matrix metalloproteinase-9 in human oral squamous cell carcinoma cells. Oncotarget (2017) 8:83088–99. doi: 10.18632/oncotarget.20236 PMC5669952 29137326

[20] Sowa T Menju T Chen-Yoshikawa TF Takahashi K Nishikawa S Nakanishi T. Hypoxia-inducible factor 1 promotes chemoresistance of lung cancer by inducing carbonic anhydrase IX expression. Cancer Med (2017) 6:288–97. doi: 10.1002/cam4.991 PMC5269694 28028936

[21] Becker HM. Carbonic anhydrase IX and acid transport in cancer. Br J Cancer (2020) 122:157–67. doi: 10.1038/s41416-019-0642-z PMC7051959 31819195

[22] Boedtkjer E Pedersen SF. The acidic tumor microenvironment as a driver of cancer. Annu Rev Physiol (2020) 82:103–26. doi: 10.1146/annurev-physiol-021119-034627 31730395

[23] Venkateswaran G Dedhar S. Interplay of carbonic anhydrase IX with amino acid and Acid/Base transporters in the hypoxic tumor microenvironment. Front Cell Dev Biol (2020) 8:602668. doi: 10.3389/fcell.2020.602668 33240897 PMC7680889

[24] McDonald PC Chia S Bedard PL Chu Q Lyle M Tang L. A phase 1 study of SLC-0111, a novel inhibitor of carbonic anhydrase IX, in patients with advanced solid tumors. Am J Clin Oncol Cancer Clin Trials (2020) 43:484–90. doi: 10.1097/COC.0000000000000691 PMC7323835 32251122

[25] Pastoreková S Závadová Z Košťál M Babušíková O Závada J. A novel quasi-viral agent, MaTu, is a two-component system. Virology (1992) 187:620–6. doi: 10.1016/0042-6822(92)90464-Z 1312272

[26] Kallio MA Tuimala JT Hupponen T Klemelä P Gentile M Scheinin I. Chipster: User-friendly analysis software for microarray and other high-throughput data. BMC Genomics (2011) 12:507. doi: 10.1186/1471-2164-12-507 21999641 PMC3215701

[27] Irizarry RA Hobbs B Collin F Beazer-Barclay YD Antonellis KJ Scherf U. Exploration, normalization, and summaries of high density oligonucleotide array probe level data. Biostatistics (2003) 4:249–64. doi: 10.1093/biostatistics/4.2.249 12925520

[28] Li C Wong WH. Model-based analysis of oligonucleotide arrays: expression index computation and outlier detection. Proc Natl Acad Sci (2001) 98:31–6. doi: 10.1073/pnas.98.1.31 PMC14539 11134512

[29] Smyth GK. Linear models and empirical bayes methods for assessing differential expression in microarray experiments. Stat Appl Genet Mol Biol (2004) 3. doi: 10.2202/1544-6115.1027 16646809

[30] Wenger R Kurtcuoglu V Scholz C Marti H Hoogewijs D. Frequently asked questions in hypoxia research. Hypoxia (2015) 3:35. doi: 10.2147/hp.s92198 27774480 PMC5045069

[31] Wagner AE Schwarzmayr T Häberle B Vokuhl C Schmid I von Schweinitz D. SP8 promotes an aggressive phenotype in hepatoblastoma *via* FGF8 activation. Cancers (Basel) (2020) 12:1–19. doi: 10.3390/cancers12082294 PMC7465460 32824198

[32] Barrett T Wilhite SE Ledoux P Evangelista C Kim IF Tomashevsky M. NCBI GEO: Archive for functional genomics data sets - update. Nucleic Acids Res (2013) 41:D991–5. doi: 10.1093/nar/gks1193 PMC3531084 23193258

[33] Andrews S. FastQC: a quality control tool for high throughput sequence data (2010). Available at: http://www.bioinformatics.babraham.ac.uk/projects/.

[34] Kim D Langmead B Salzberg SL. HISAT: A fast spliced aligner with low memory requirements. Nat Methods (2015) 12:357–60. doi: 10.1038/nmeth.3317 PMC4655817 25751142

[35] Anders S Pyl PT Huber W. HTSeq-a Python framework to work with high-throughput sequencing data. Bioinformatics (2015) 31:166–9. doi: 10.1093/bioinformatics/btu638 PMC4287950 25260700

[36] Robinson MD McCarthy DJ Smyth GK. edgeR: A bioconductor package for differential expression analysis of digital gene expression data. Bioinformatics (2009) 26:139–40. doi: 10.1093/bioinformatics/btp616 PMC2796818 19910308

[37] Chen EY Tan CM Kou Y Duan Q Wang Z Meirelles GV. Enrichr: Interactive and collaborative HTML5 gene list enrichment analysis tool. BMC Bioinf (2013) 14:128. doi: 10.1186/1471-2105-14-128 PMC3637064 23586463

[38] Kuleshov MV Jones MR Rouillard AD Fernandez NF Duan Q Wang Z. Enrichr: a comprehensive gene set enrichment analysis web server 2016 update. Nucleic Acids Res (2016) 44:W90–7. doi: 10.1093/nar/gkw377 PMC4987924 27141961

[39] Livak KJ Schmittgen TD. Analysis of relative gene expression data using real-time quantitative PCR and the 2-ΔΔCT method. Methods (2001) 25:402–8. doi: 10.1006/meth.2001.1262 11846609

[40] Daina A Michielin O Zoete V. SwissTargetPrediction: updated data and new features for efficient prediction of protein targets of small molecules. Nucleic Acids Res (2019) 47:W357–W3664. doi: 10.1093/nar/gkz382 31106366 PMC6602486

[41] Nickel J Gohlke BO Erehman J Banerjee P Rong WW Goede A. SuperPred: Update on drug classification and target prediction. Nucleic Acids Res (2014) 42:W26–31. doi: 10.1093/nar/gku477 PMC4086135 24878925

[42] Hirschhaeuser F Menne H Dittfeld C West J Mueller-Klieser W Kunz-Schughart LA. Multicellular tumor spheroids: An underestimated tool is catching up again. J Biotechnol (2010) 148:3–15. doi: 10.1016/j.jbiotec.2010.01.012 20097238

[43] Ciccone V Filippelli A Angeli A Supuran CT Morbidelli L. Pharmacological inhibition of CA-IX impairs tumor cell proliferation, migration and invasiveness. Int J Mol Sci (2020) 21:e. doi: 10.3390/ijms21082983 PMC7215745 32340282

[44] Shin HJ Rho SB Jung DC Han IO Oh ES Kim JY. Carbonic anhydrase IX (CA9) modulates tumorassociated cell migration and invasion. J Cell Sci (2011) 124:1077–87. doi: 10.1242/jcs.072207 21363891

[45] Güttler A Theuerkorn K Riemann A Wichmann H Kessler J Thews O. Cellular and radiobiological effects of carbonic anhydrase IX in human breast cancer cells. Oncol Rep (2019) 41:2585–94. doi: 10.3892/or.2019.7001 30720123

[46] Kaluz S Kaluzová M Liao SY Lerman M Stanbridge EJ. Transcriptional control of the tumor- and hypoxia-marker carbonic anhydrase 9: A one transcription factor (HIF-1) show? Biochim Biophys Acta - Rev Cancer (2009) 1795:162–72. doi: 10.1016/j.bbcan.2009.01.001 PMC2670353 19344680

[47] Rhee H Nahm JH Kim H Choi GH Yoo JE Lee HS. Poor outcome of hepatocellular carcinoma with stemness marker under hypoxia: Resistance to transarterial chemoembolization. Mod Pathol (2016) 29:1038–49. doi: 10.1038/modpathol.2016.111 27312064

[48] Huang WJ Jeng YM Lai HS Fong IU Sheu FYB Lai PL. Expression of hypoxic marker carbonic anhydrase IX predicts poor prognosis in resectable hepatocellular carcinoma. PloS One (2015) 10:e0119181–e0119181. doi: 10.1371/journal.pone.0119181 25738958 PMC4349857

[49] Plaks V Kong N Werb Z. The cancer stem cell niche: How essential is the niche in regulating stemness of tumor cells? Cell Stem Cell (2015) 16:225–38. doi: 10.1016/j.stem.2015.02.015 PMC4355577 25748930

[50] Marie-Egyptienne DT Chaudary N Kalliomäki T Hedley DW Hill RP. Cancer initiating-cells are enriched in the CA9 positive fraction of primary cervix cancer xenografts. Oncotarget (2017) 8:1392–404. doi: 10.18632/oncotarget.13625 PMC5352063 27901496

[51] Lock FE McDonald PC Lou Y Serrano I Chafe SC Ostlund C. Targeting carbonic anhydrase IX depletes breast cancer stem cells within the hypoxic niche. Oncogene (2013) 32:5210–9. doi: 10.1038/onc.2012.550 23208505

[52] Sørensen BS Hao J Overgaard J Vorum H Honoré B Alsner J. Influence of oxygen concentration and pH on expression of hypoxia induced genes. Radiother Oncol (2005) 76:187–93. doi: 10.1016/j.radonc.2005.06.037 16098620

[53] Proescholdt MA Merrill MJ Stoerr EM Lohmeier A Pohl F Brawanski A. Function of carbonic anhydrase IX in glioblastoma multiforme. Neuro Oncol (2012) 14:1357–66. doi: 10.1093/neuonc/nos216 PMC3480266 23074198

[54] Sáenz-de-Santa-María I Bernardo-Castiñeira C Secades P Bernaldo-de-Quirós S Rodrigo JP Astudillo A. Clinically relevant HIF-1α-dependent metabolic reprogramming in oropharyngeal squamous cell carcinomas includes coordinated activation of CAIX and the miR-210/ISCU signaling axis, but not MCT1 and MCT4 upregulation. Oncotarget (2017) 8:13730–46. doi: 10.18632/oncotarget.14629 PMC5355133 28099149

[55] Chen CL Chu JS Su WC Huang SC Lee WY. Hypoxia and metabolic phenotypes during breast carcinogenesis: Expression of HIF-1α, GLUT1, and CAIX. Virchows Arch (2010) 457:53–61. doi: 10.1007/s00428-010-0938-0 20526721

[56] Kuchuk O Tuccitto A Citterio D Huber V Camisaschi C Milione M. pH regulators to target the tumor immune microenvironment in human hepatocellular carcinoma. Oncoimmunology (2018) 7:e1445452. doi: 10.1080/2162402X.2018.1445452 29900055 PMC5993489

[57] Lou Y McDonald PC Oloumi A Chia S Ostlund C Ahmadi A. Targeting tumor hypoxia: Suppression of breast tumor growth and metastasis by novel carbonic anhydrase IX inhibitors. Cancer Res (2011) 71:3364–76. doi: 10.1158/0008-5472.CAN-10-4261 21415165

[58] Chafe SC McDonald PC Saberi S Nemirovsky O Venkateswaran G Burugu S. Targeting hypoxia-induced carbonic anhydrase IX enhances immune-checkpoint blockade locally and systemically. Cancer Immunol Res (2019) 7:1064–78. doi: 10.1158/2326-6066.CIR-18-0657 31088846

[59] Angeli A Carta F Nocentini A Winum JY Zalubovskis R Akdemir A. Carbonic anhydrase inhibitors targeting metabolism and tumor microenvironment. Metabolites (2020) 10:1–21. doi: 10.3390/metabo10100412 PMC7602163 33066524

[60] Al-Warhi T Elbadawi MM Bonardi A Nocentini A Al-Karmalawy AA Aljaeed N. Design and synthesis of benzothiazole-based SLC-0111 analogues as new inhibitors for the cancer-associated carbonic anhydrase isoforms IX and XII. J Enzyme Inhib Med Chem (2022) 37:2635–43. doi: 10.1080/14756366.2022.2124409 PMC9518259 36146927

[61] Krasavin M Kalinin S Sharonova T Supuran CT. Inhibitory activity against carbonic anhydrase IX and XII as a candidate selection criterion in the development of new anticancer agents. J Enzyme Inhib Med Chem (2020) 35:1555–61. doi: 10.1080/14756366.2020.1801674 PMC7470080 32746643

[62] Yang JS Lin CW Chuang CY Su SC Lin SH Yang SF. Carbonic anhydrase IX overexpression regulates the migration and progression in oral squamous cell carcinoma. Tumor Biol (2015) 36:9517–24. doi: 10.1007/s13277-015-3692-8 26130414

[63] Swayampakula M McDonald PC Vallejo M Coyaud E Chafe SC Westerback A. The interactome of metabolic enzyme carbonic anhydrase IX reveals novel roles in tumor cell migration and invadopodia/MMP14-mediated invasion. Oncogene (2017) 36:6244–61. doi: 10.1038/onc.2017.219 PMC5684442 28692057

[64] Debreova M Csaderova L Burikova M Lukacikova L Kajanova I Sedlakova O. CAIX regulates invadopodia formation through both a pH-dependent mechanism and interplay with actin regulatory proteins. Int J Mol Sci (2019) 20(11):2745. doi: 10.3390/ijms20112745 31167468 PMC6600150

[65] Drenckhan A Freytag M Supuran CT Sauter G Izbicki JR Gros SJ. CAIX furthers tumour progression in the hypoxic tumour microenvironment of esophageal carcinoma and is a possible therapeutic target. J Enzyme Inhib Med Chem (2018) 33:1024–33. doi: 10.1080/14756366.2018.1475369 PMC6010094 29865880

[66] Hsieh MJ Chen KS Chiou HL Hsieh YS. Carbonic anhydrase XII promotes invasion and migration ability of MDA-MB-231 breast cancer cells through the p38 MAPK signaling pathway. Eur J Cell Biol (2010) 89:598–606. doi: 10.1016/j.ejcb.2010.03.004 20434230

[67] Švastová E Žilka N Zat’ovičová M Gibadulinová A Čiampor F Pastorek J. Carbonic anhydrase IX reduces e-cadherin-mediated adhesion of MDCK cells *via* interaction with β-catenin. Exp Cell Res (2003) 290:332–45. doi: 10.1016/S0014-4827(03)00351-3 14567991

[68] Svastova E Witarski W Csaderova L Kosik I Skvarkova L Hulikova A. Carbonic anhydrase IX interacts with bicarbonate transporters in lamellipodia and increases cell migration *via* its catalytic domain. J Biol Chem (2012) 287:3392–402. doi: 10.1074/jbc.M111.286062 PMC3270993 22170054

[69] Congiu C Onnis V Deplano A Balboni G Dedeoglu N Supuran CT. Synthesis of sulfonamides incorporating piperazinyl-ureido moieties and their carbonic anhydrase I, II, IX and XII inhibitory activity. Bioorg Med Chem Lett (2015) 25:3850–3. doi: 10.1016/j.bmcl.2015.07.060 26233435

[70] Ruzzolini J Laurenzana A Andreucci E Peppicelli S Bianchini F Carta F. A potentiated cooperation of carbonic anhydrase IX and histone deacetylase inhibitors against cancer. J Enzyme Inhib Med Chem (2020) 35:391–7. doi: 10.1080/14756366.2019.1706090 PMC6968260 31865754

[71] He L Cai X Cheng S Zhou H Zhang Z Ren J. Ornithine transcarbamylase downregulation is associated with poor prognosis in hepatocellular carcinoma. Oncol Lett (2019) 17:5030–8. doi: 10.3892/ol.2019.10174 PMC6507468 31186714

[72] Sakamoto LHT De Camargo B Cajaiba M Soares FA Vettore AL. MT1G hypermethylation: A potential prognostic marker for hepatoblastoma. Pediatr Res (2010) 67:387–93. doi: 10.1203/PDR.0b013e3181d01863 20032811

[73] Takata A Otsuka M Yoshikawa T Kishikawa T Hikiba Y Obi S. MicroRNA-140 acts as a liver tumor suppressor by controlling NF-κB activity by directly targeting DNA methyltransferase 1 (Dnmt1) expression. Hepatology (2013) 57:162–70. doi: 10.1002/hep.26011 PMC3521841 22898998

[74] Sekiguchi M Seki M Kawai T Yoshida K Yoshida M Isobe T. Integrated multiomics analysis of hepatoblastoma unravels its heterogeneity and provides novel druggable targets. NPJ Precis Oncol (2020) 4:1–12. doi: 10.1038/s41698-020-0125-y 32656360 PMC7341754

[75] Yang Z Feng J Xiao L Chen X Yao Y Li Y. Tumor-derived peptidoglycan recognition protein 2 predicts survival and antitumor immune responses in hepatocellular carcinoma. Hepatology (2020) 71:1626–42. doi: 10.1002/hep.30924 PMC7318564 31479523

[76] Xu D Wu J Dong L Luo W Li L Tang D. Serpinc1 acts as a tumor suppressor in hepatocellular carcinoma through inducing apoptosis and blocking macrophage polarization in an ubiquitin-proteasome manner. Front Oncol (2021) 11:738607. doi: 10.3389/fonc.2021.738607 34881176 PMC8645897

[77] Li Z Kwon SM Li D Li L Peng X Zhang J. Human constitutive androstane receptor represses liver cancer development and hepatoma cell proliferation by inhibiting erythropoietin signaling. J Biol Chem (2022) 298(5):101885. doi: 10.1016/j.jbc.2022.101885 35367211 PMC9052153

[78] Chen HW Huang XD Li HC He S Ni RZ Chen CH. Expression of FOXJ1 in hepatocellular carcinoma: Correlation with patients’ prognosis and tumor cell proliferation. Mol Carcinog (2013) 52:647–59. doi: 10.1002/mc.21904 22488567

[79] McDonald PC Chafe SC Supuran CT Dedhar S. Cancer therapeutic targeting of hypoxia induced carbonic anhydrase IX: From bench to bedside. Cancers (Basel) (2022) 14(14):3297. doi: 10.3390/cancers14143297 35884358 PMC9322110

