# Supplementary material for: SLC-0111, an inhibitor of carbonic anhydrase IX, attenuates hepatoblastoma cell viability and migration
Source: Front Oncol. 2023 Jan 26;13:1118268. doi: 10.3389/fonc.2023.1118268 (PMC9909558; doi:10.3389/fonc.2023.1118268)
Supplement: Supplementary file 1 [file DataSheet_1.pdf]

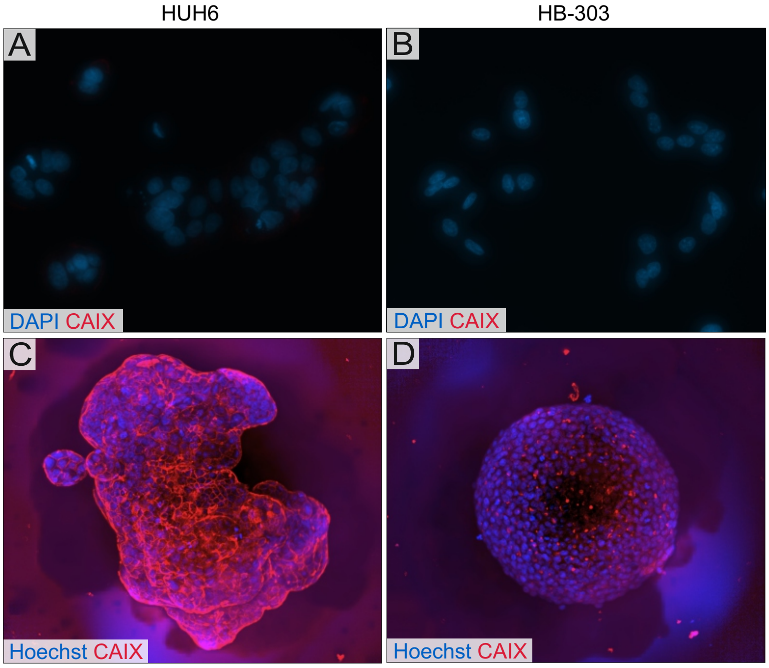

Supplementary figure 1. CAIX is expressed in HB cells and spheroids. CAIX expression was negligible in HUH6 (A) and HB-303 (B) cells while in spheroids (C and D) the staining intensity was stronger.
