# Supplementary material for: SLC-0111, an inhibitor of carbonic anhydrase IX, attenuates hepatoblastoma cell viability and migration
Source: Front Oncol. 2023 Jan 26;13:1118268. doi: 10.3389/fonc.2023.1118268 (PMC9909558; doi:10.3389/fonc.2023.1118268)
Supplement: Supplementary file 2 [file DataSheet_2.pdf]

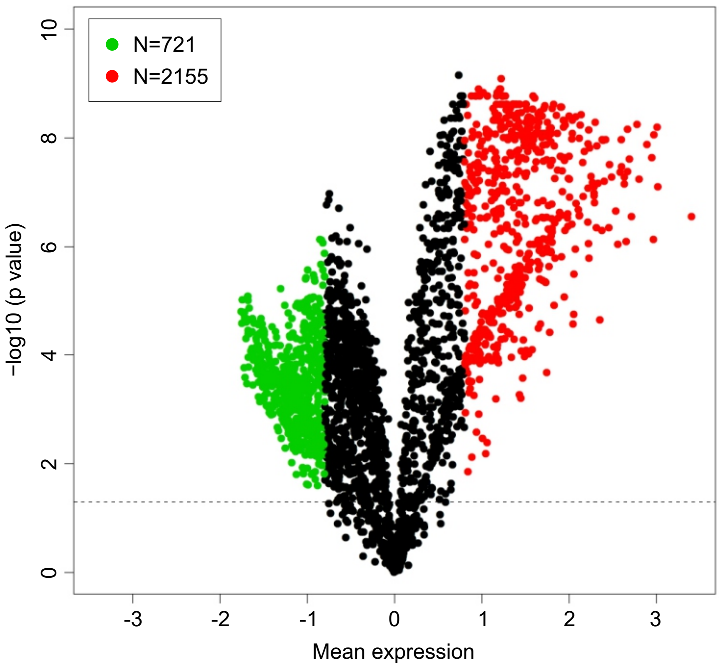

Supplementary figure 2. Volcanoplot of differential gene expression of HUH6 cells in hypoxia vs. normoxia. Log2 fold change of 0.8 and adjusted p-value 0.05 were concidered as significant.
